# Case Report: A longitudinal single-patient observation of metabolic changes during thyrotoxicosis due to toxic multinodular goiter in type 2 diabetes

**DOI:** 10.3389/fendo.2026.1989874

**Published:** 2026-10-27

**Authors:** Hussein A. Rajab

**Affiliations:** 1 Department of Internal Medicine–Endocrinology, College of Medicine, Najran University, Najran, Saudi Arabia; 2 Health Research Center, Najran University, Najran, Saudi Arabia

**Keywords:** hyperglycemia, hypertension, low-density lipoprotein cholesterol, thyroidectomy, thyrotoxicosis, toxic multinodular goiter, type 2 diabetes mellitus

## Abstract

Thyrotoxicosis can worsen glucose regulation and is also associated with lower circulating total cholesterol (TC) and low-density lipoprotein cholesterol (LDL-C), a metabolic pattern that may be less readily recognized in routine diabetes care. We report a woman aged 51 years at first presentation, with approximately 20 years of type 2 diabetes mellitus and previously diagnosed thyrotoxicosis due to toxic multinodular goiter, who self-referred to our endocrine clinic on September 10, 2024 seeking help with poor glycemic control before planned thyroid surgery. A recent glycated hemoglobin (HbA1c) was 11.8% (105 mmol/mol), she had lost approximately 17 kg, and she reported marked hunger, frequent snacking, heat intolerance, sweating, tremor, insomnia, irritability, palpitations, polyuria, and polydipsia. Dietary contributors were addressed, glimepiride was replaced with pioglitazone, and metformin was increased. Before basal insulin was introduced, fasting plasma glucose (FPG) and HbA1c improved to 183.4 mg/dL (10.2 mmol/L) and 8.9% (74 mmol/mol) on May 6, 2025 while thyroid hormone concentrations also improved. When thyrotoxicosis subsequently worsened on September 15, 2025, free triiodothyronine (FT3) was 15.5 pmol/L and free thyroxine (FT4) was 3.8 ng/dL (48.9 pmol/L), FPG rose to 293.7 mg/dL (16.3 mmol/L) and HbA1c to 12.1% (109 mmol/mol), and TC and directly measured LDL-C were 121.9 mg/dL (3.2 mmol/L) and 45 mg/dL (1.2 mmol/L), respectively. With continued antithyroid treatment and later intensification of diabetes therapy, thyroid biochemistry and glycemia improved; by August 24, 2026, FT3 was 6.1 pmol/L, FT4 1.5 ng/dL (19.3 pmol/L), FPG 117.8 mg/dL (6.5 mmol/L), HbA1c 9.4% (79 mmol/mol), TC 151.4 mg/dL (3.9 mmol/L), and LDL-C 66 mg/dL (1.7 mmol/L). The patient preferred thyroidectomy and developed compressive symptoms, but surgery remained delayed because of glycemic concerns and long surgical appointment intervals. This longitudinal pattern suggests that thyrotoxicosis may have contributed to glycemic instability and the low LDL-C and TC pattern. Concurrent diabetes-treatment changes prevent attribution to thyroid status alone, but the case highlights that behavioral contributors and physiological drivers can coexist and that thyrotoxicosis should be considered when metabolic deterioration appears disproportionate to behavioral factors.

## 1 Introduction

Diabetes mellitus and thyroid disorders frequently coexist, and thyroid hormone excess can substantially alter glucose metabolism. Hyperthyroidism increases hepatic glucose production through gluconeogenesis and glycogenolysis, increases intestinal glucose absorption and lipolysis, alters insulin sensitivity, and accelerates insulin degradation and clearance. It may also increase insulin demand and alter pancreatic beta-cell secretory dynamics, effects that can be especially important in long-standing type 2 diabetes with limited beta-cell reserve (1). Thyrotoxicosis also commonly causes weight loss despite normal or increased appetite, together with heat intolerance, sweating, tremor, palpitations, anxiety, and disturbed sleep (2). In a patient with diabetes, this combination of altered glucose metabolism and a physiologically increased drive to eat can further complicate glycemic control.

Thyroid hormone excess also has a characteristic effect on lipid metabolism. Increased hepatic low-density lipoprotein receptor activity enhances clearance of low-density lipoprotein particles, and human studies suggest that thyroid hormone may reduce circulating proprotein convertase subtilisin/kexin type 9 (PCSK9), further favoring their removal (3). A systematic review and meta-analysis found that total cholesterol (TC) and low-density lipoprotein cholesterol (LDL-C) generally rise after treatment of overt hyperthyroidism (4).

This report describes a 23-month longitudinal course in which changes in thyrotoxic symptoms and thyroid biochemistry occurred alongside marked changes in glycemic control and lipid concentrations in a woman with long-standing type 2 diabetes and toxic multinodular goiter. The case also illustrates the practical challenge of controlling both conditions while poor glycemic control delayed the patient’s preferred definitive thyroid surgery.

## 2 Case description

### 2.1 Patient information and clinical presentation

A woman aged 51 years at her first clinic visit (53 years by the end of follow-up), with type 2 diabetes mellitus diagnosed around 2004, self-referred to our endocrine clinic on September 10, 2024 seeking assistance with poor glycemic control before planned thyroid surgery. She had already been diagnosed with thyrotoxicosis due to toxic multinodular goiter at another hospital after abnormal thyroid-function tests and radionuclide thyroid imaging. She continued follow-up with a surgical team at that hospital, but elective thyroidectomy had been deferred because her diabetes remained poorly controlled and the team requested improvement to a “reasonable” glycated hemoglobin (HbA1c) before proceeding. No specific numerical HbA1c threshold was documented. A recent HbA1c measured on August 27, 2024 was 11.8% (105 mmol/mol).

At presentation, she described fatigue, excessive sweating, increased appetite and hunger, heat intolerance, irritability, anxiety, severe insomnia, exertional palpitations, dyspnea on exertion, polyuria, polydipsia, increased bowel frequency, and fine hand tremor. She reported an approximately 17-kg weight loss, from 94 to 77 kg. During symptomatic periods she could remain awake for 2–3 days and described marked restlessness and emotional lability. Compressive symptoms were not prominent initially but emerged later during follow-up.

She acknowledged difficulty adhering to dietary recommendations at the initial visit, including frequent snacking and regular intake of white bread, added sugar, and soft drinks. Exercise was limited by leg pain. There was no reported family history of thyroid disease, diabetes mellitus, or autoimmune disease. Diabetes complications included pre-proliferative diabetic retinopathy, with no documented diabetic neuropathy or nephropathy.

On examination, she appeared restless and emotional. Blood pressure was 138/85 mmHg and pulse rate was 86 beats/min. A fine hand tremor was present. Thyroid examination showed mild diffuse enlargement without an audible thyroid bruit. There was no lid lag, lid retraction, or exophthalmos, and Pemberton’s sign was negative.

### 2.2 Diagnostic assessment

The prior diagnosis of thyrotoxicosis due to toxic multinodular goiter was supported by biochemical thyroid dysfunction and radionuclide thyroid imaging. The available technetium-99m (Tc-99m) pertechnetate thyroid uptake scan report described an enlarged gland with heterogeneous uptake and at least two focal areas of increased uptake in the upper and lower poles of the left lobe, suggestive of hyperfunctioning (hot) nodules. The calculated Tc-99m uptake was 5.5%. The report concluded that the findings were suggestive of toxic multinodular goiter (Plummer disease), with Graves disease considered a less likely differential diagnosis. Serial testing during follow-up in our clinic repeatedly showed suppressed thyroid-stimulating hormone (TSH) with elevated free triiodothyronine (FT3) and/or free thyroxine (FT4). Laboratory reference ranges were TSH 0.270-4.20 mIU/L, FT3 3.1-6.8 pmol/L, and FT4 0.9-1.7 ng/dL (11.9-21.6 pmol/L).

All longitudinal fasting plasma glucose (FPG) measurements reported in this case were obtained in the clinical laboratory during routine follow-up. The patient also performed intermittent capillary glucose monitoring at home and reported that readings were frequently elevated during periods of poor control; however, systematic meter downloads, continuous glucose monitoring, and complete home glucose logs were not available.

### 2.3 Therapeutic intervention

At the initial visit, diabetes treatment included metformin 850 mg twice daily, empagliflozin 25 mg once daily, sitagliptin 100 mg once daily, and glimepiride 6 mg daily. Glimepiride was discontinued and replaced with pioglitazone 15 mg daily. While this change would not necessarily be expected to produce greater glucose lowering pharmacologically, reducing sulfonylurea exposure may have lessened episodes of possible hypoglycemia and hunger, which in turn may have reduced excessive snacking and unhealthy carbohydrate intake. Dietary counseling emphasizing reduction of refined carbohydrates and added sugars was reinforced, and metformin was subsequently increased to 1000 mg twice daily while empagliflozin and sitagliptin were continued.

Because glycemic control later remained inadequate, basal insulin glargine was introduced on January 1, 2026 at 7 units daily, increased to 10 units by February, and subsequently titrated to 16 units and then 25 units daily. A documented titration instruction advised increasing glargine by 2 units every few days toward an FPG target of 80–120 mg/dL (4.4-6.7 mmol/L).

Thyrotoxicosis was treated with carbimazole and propranolol. Carbimazole doses ranged from 5 mg twice daily to 10 mg three times daily according to symptoms and thyroid biochemistry. Propranolol was prescribed within symptom-guided dose ranges, and the patient was instructed to adjust the dose within the prescribed range according to palpitations, tremor, and other adrenergic symptoms. Documented propranolol doses ranged from 10 mg twice daily to 20–40 mg two or three times daily during more symptomatic periods. Liver biochemistry was followed during carbimazole therapy as part of clinical care.

The patient preferred thyroidectomy as definitive treatment and continued follow-up with the surgical team at the other hospital. During this period, our clinic managed both diabetes and thyrotoxicosis. Surgery remained delayed because satisfactory glycemic control had not yet been achieved and because surgical appointments were widely spaced. The later development of compressive symptoms, including dysphagia, hoarseness, shortness of breath, and a choking sensation, reinforced the need for definitive treatment. Thyroidectomy had not been performed by the last documented follow-up. The longitudinal clinical findings, laboratory measurements, and treatment changes are summarized in **Table 1**.

**Table 1:** Longitudinal clinical and laboratory timeline, September 10, 2024 to August 24, 2026.

Date	Symptoms/clinical course	BP (mmHg)	TSH (mIU/L)	FT3 (pmol/L)	FT4 ng/dL (pmol/L)	FPG/HbA1c	TC/LDL-C	Thyroid treatment/change	Diabetes treatment/change
10 Sep 2024	Fatigue, sweating, hunger, heat intolerance, irritability/anxiety, severe insomnia, palpitations, dyspnea, polyuria/polydipsia, tremor; ~17-kg weight loss	138/85	0.005	5.5	2.3 (29.6)	NA/11.8% (105)	125.7 (3.3)/38.7 (1.0)	Not recorded	Baseline: metformin 850 mg BID, empagliflozin 25 mg QD, sitagliptin 100 mg QD, glimepiride 6 mg QD
26 Nov 2024	Persistent fatigue, sweating, hunger, irritability, insomnia, heat intolerance, exertional dyspnea/palpitations, tremor	110/70	0.005	11.6	3.3 (42.5)	306.5 (17.0)/11.1% (98)	121.5 (3.1)/43 (1.1)	Carbimazole 5 mg BID; propranolol 10 mg BID	Glimepiride stopped; pioglitazone 15 mg QD started; metformin 1000 mg BID
6 May 2025	—	130/80	0.005	8.6	1.9 (24.5)	183.4 (10.2)/8.9% (74)	146.8 (3.8)/55 (1.4)	No change	No change
15 Sep 2025	Polyuria, severe sleep disturbance, heat intolerance, hyperdefecation, residual palpitations; anxiety/irritability and hunger improved; dysphagia, hoarseness, dyspnea	160/90	0.005	15.5	3.8 (48.9)	293.7 (16.3)/12.1% (109)	121.9 (3.2)/45 (1.2)	Carbimazole 5 mg TID; propranolol 10 mg TID	No change
29 Sep 2025	Irritability/anxiety improved; persistent severe insomnia, residual palpitations, less hunger, polyuria	120/76	0.005	NA	NA	273.2 (15.2)/12.4% (112)	145.4 (3.8)/58 (1.5)	Carbimazole 10 mg TID; propranolol 20–40 mg TID	No change
14 Oct 2025	Less heat intolerance, tremor, palpitations and hyperdefecation; anxiety improved	110/70	0.000	6.5	NA	NA	NA	Carbimazole 10 mg TID; propranolol 20–40 mg BID	No change
18 Nov 2025	Less palpitations, irritability, appetite, polyuria/polydipsia and fatigue; no hyperdefecation; choking improved but persisted	124/78	0.010	6.4	NA	192.1 (10.7)/11.1% (98)	149.7 (3.9)/65.4 (1.7)	Carbimazole 10 mg BID; propranolol 20 mg BID	No change
1 Jan 2026	—	130/80	0.005	7.5	1.9 (24.5)	232.3 (12.9)/10.3% (89)	147.3 (3.8)/63 (1.6)	No change	Insulin glargine 7 U daily started
10 Feb 2026	No palpitations; less anxiety, tremor and sweating; sleeping well	128/72	0.006	5.6	1.5 (19.3)	185.8 (10.3)/10.5% (91)	146.0 (3.8)/72 (1.9)	No change	Insulin glargine increased to 10 U daily
6 Apr 2026	—	NA	0.019	5.4	1.3 (16.7)	219.4 (12.2)/9.4% (79)	153.5 (4.0)/54 (1.4)	Propranolol reduced to 10 mg BID; carbimazole 10 mg BID	Glargine 10 U; titration by 2 U every few days toward FPG 80–120 mg/dL (4.4-6.7 mmol/L)
4 Jun 2026	—	140/70	0.005	10.2	2.4 (30.9)	295.9 (16.4)/10.4% (90)	135.2 (3.5)/51 (1.3)	Carbimazole increased to 10 mg TID; propranolol 10 mg BID	Glargine 16 U daily
16 Jul 2026	—	NA	0.005	9.2	2.3 (29.6)	226.3 (12.6)/10.3% (89)	134.8 (3.5)/52 (1.3)	No change	Glargine 25 U daily
24 Aug 2026	Less tremor, palpitations, irritability and sweating	130/76	NA	6.1	1.5 (19.3)	117.8 (6.5)/9.4% (79)	151.4 (3.9)/66 (1.7)	No change	No change

Reference ranges: TSH 0.270-4.20 mIU/L; FT3 3.1-6.8 pmol/L; FT4 0.9-1.7 ng/dL (11.9-21.6 pmol/L). BP, blood pressure; TSH, thyroid-stimulating hormone; FT3, free triiodothyronine; FT4, free thyroxine; FPG, fasting plasma glucose; HbA1c, glycated hemoglobin; TC, total cholesterol; LDL-C, low-density lipoprotein cholesterol; NA, not available in the medical record; BID, twice daily; TID, three times daily; QD, once daily; U, units. For paired laboratory values, conventional units are shown first and SI equivalents in parentheses: FPG, mg/dL (mmol/L); HbA1c, % (mmol/mol); FT4, ng/dL (pmol/L); TC and directly measured LDL-C, mg/dL (mmol/L). FT3 and TSH are reported in SI units only. Missing values were not imputed. Propranolol dose ranges were symptom-guided.

### 2.4 Follow-up and outcomes

Symptoms and biochemical results fluctuated over the 23-month observation period. On September 15, 2025, the patient still reported marked sleep disturbance, polyuria, heat intolerance, hyperdefecation, and residual palpitations despite some improvement in anxiety, irritability, and hunger. Dysphagia, hoarseness, shortness of breath, and other compressive symptoms were also documented. By November 18, 2025, palpitations, irritability, appetite, polyuria, polydipsia, fatigue, and bowel frequency had improved; a choking sensation had decreased but remained present. By February 10, 2026, she reported sleeping well, no palpitations, and less anxiety, tremor, and sweating. At the final documented visit on August 24, 2026, tremor, palpitations, irritability, and sweating were again improved.

The glycemic course showed repeated changes alongside thyroid activity. On November 26, 2024, FPG was 306.5 mg/dL (17.0 mmol/L) and HbA1c 11.1% (98 mmol/mol) while FT3 was 11.6 pmol/L and FT4 3.3 ng/dL (42.5 pmol/L). By May 6, 2025 - before basal insulin was introduced - FT3 had fallen to 8.6 pmol/L and FT4 to 1.9 ng/dL (24.5 pmol/L), while FPG improved to 183.4 mg/dL (10.2 mmol/L) and HbA1c to 8.9% (74 mmol/mol). When thyrotoxicosis worsened on September 15, 2025, FT3 rose to 15.5 pmol/L and FT4 to 3.8 ng/dL (48.9 pmol/L), with FPG 293.7 mg/dL (16.3 mmol/L) and HbA1c 12.1% (109 mmol/mol). On June 4, 2026, FT3 was 10.2 pmol/L and FT4 2.4 ng/dL (30.9 pmol/L), with FPG 295.9 mg/dL (16.4 mmol/L) and HbA1c 10.4% (90 mmol/mol). By August 24, 2026, FT3 had fallen to 6.1 pmol/L and FT4 to 1.5 ng/dL (19.3 pmol/L), while FPG was 117.8 mg/dL (6.5 mmol/L) and HbA1c 9.4% (79 mmol/mol) (**Figure 1**).

**Figure 1:**
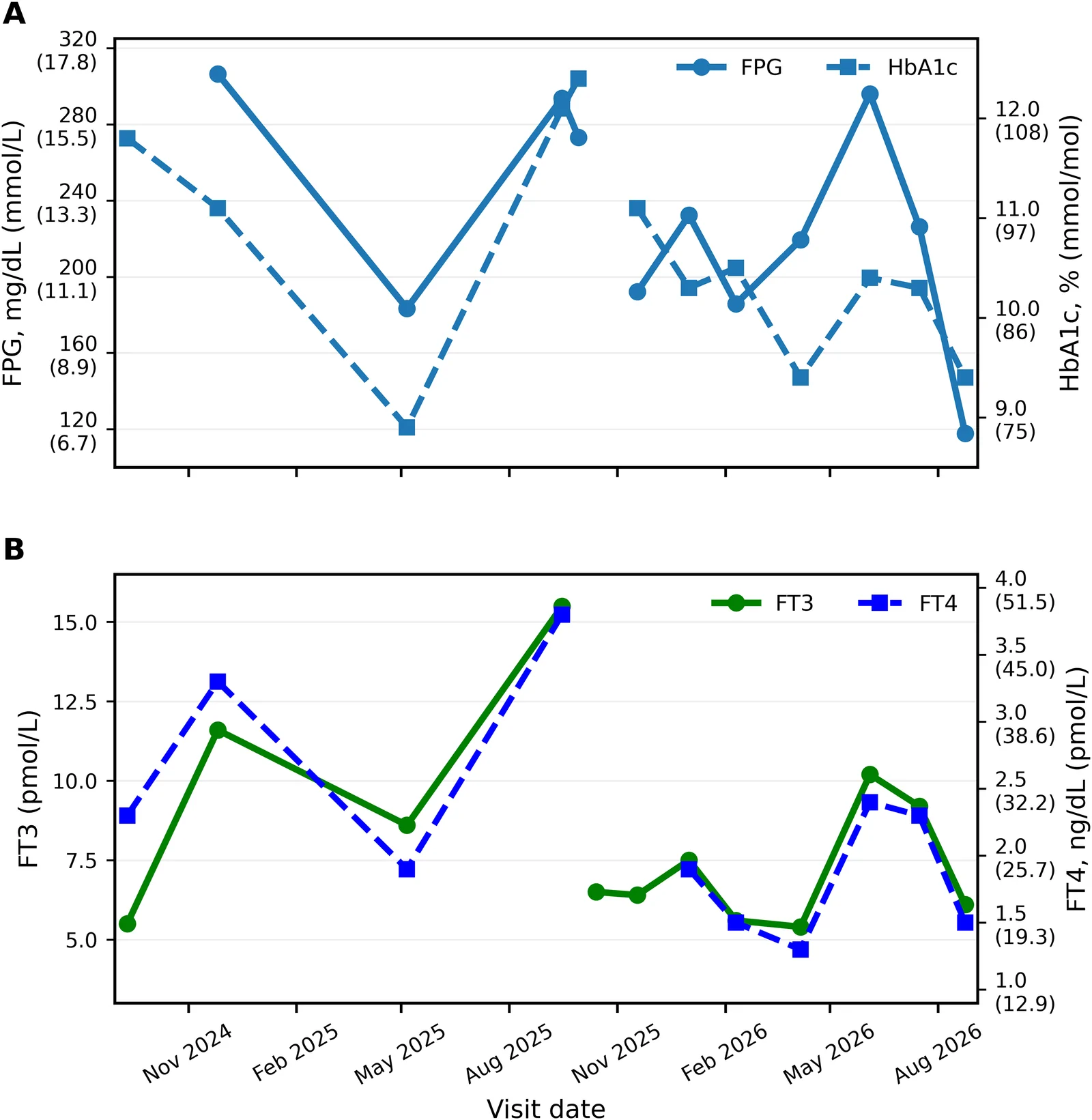
Longitudinal glycemic and thyroid measurements during follow-up. **(A)** Fasting plasma glucose (FPG; n=11) and glycated hemoglobin (HbA1c; n=12). **(B)** Free triiodothyronine (FT3; n=12) and free thyroxine (FT4; n=10). Conventional units are shown first, with SI equivalents in parentheses where applicable: FPG, mg/dL (mmol/L); HbA1c, % (mmol/mol); FT3, pmol/L; FT4, ng/dL (pmol/L). Measurements are plotted by actual clinic-visit date from September 2024 through August 2026. All available measurements are shown; missing values were not imputed. Thyroid laboratory reference ranges were FT3 3.1-6.8 pmol/L and FT4 0.9-1.7 ng/dL (11.9-21.6 pmol/L). n, number of available measurements.

The lipid profile showed a parallel longitudinal pattern. The earliest available values were TC 125.7 mg/dL (3.3 mmol/L) and directly measured LDL-C 38.7 mg/dL (1.0 mmol/L). The patient was not receiving a statin or other lipid-lowering medication during follow-up. On September 15, 2025, TC was 121.9 mg/dL (3.2 mmol/L) and LDL-C 45 mg/dL (1.2 mmol/L); on June 4, 2026, they were 135.2 mg/dL (3.5 mmol/L) and 51 mg/dL (1.3 mmol/L); and on August 24, 2026, they were 151.4 mg/dL (3.9 mmol/L) and 66 mg/dL (1.7 mmol/L), respectively (**Figure 2**).

**Figure 2:**
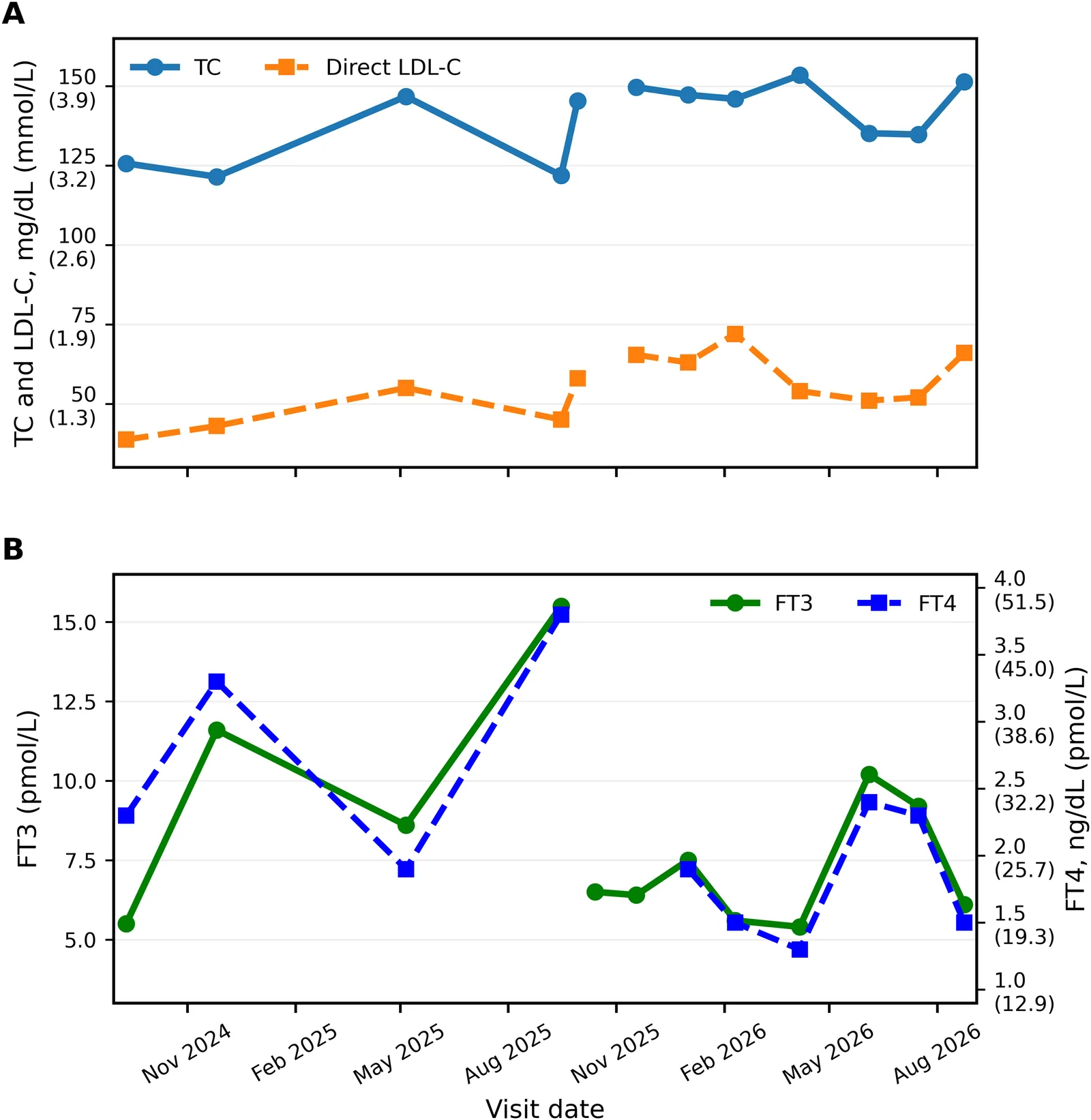
Longitudinal lipid and thyroid measurements during follow-up. **(A)** Total cholesterol (TC; n=12) and directly measured low-density lipoprotein cholesterol (LDL-C; n=12). **(B)** Free triiodothyronine (FT3; n=12) and free thyroxine (FT4; n=10). Conventional units are shown first, with SI equivalents in parentheses where applicable: TC and LDL-C, mg/dL (mmol/L); FT3, pmol/L; FT4, ng/dL (pmol/L). Measurements are plotted by actual clinic-visit date. All available measurements are shown; missing values were not imputed. The patient was not receiving statins or other lipid-lowering therapy during the observation period. n, number of available measurements.

Blood pressure also varied during follow-up, reaching 160/90 mmHg during marked biochemical thyrotoxicosis on September 15, 2025 and subsequently declining on later visits. Carbimazole was clinically tolerated. Serial alanine aminotransferase values ranged from 18.2 to 36.6 U/L (0.3-0.6 µkat/L) and aspartate aminotransferase values from 12.2 to 23.1 U/L (0.2-0.4 µkat/L); alkaline phosphatase and bilirubin remained within the laboratory reference range, with no biochemical evidence of hepatocellular or cholestatic injury. Thyroidectomy had not been performed by August 24, 2026 and remained pending at the time of manuscript revision in September 2026.

## 3 Discussion

### 3.1 Thyrotoxicosis and the glycemic course

Thyrotoxicosis can worsen glucose metabolism through increased hepatic glucose output, enhanced intestinal glucose absorption, increased lipolysis, altered insulin sensitivity, and accelerated insulin turnover (1). In a patient with long-standing type 2 diabetes and limited beta-cell reserve, these effects can increase the difficulty of maintaining glycemic control. Increased appetite is also a recognized manifestation of thyrotoxicosis, and hyperthyroidism can increase food intake despite ongoing weight loss (1, 2).

At the first visit, several behavioral factors plausibly contributed to poor control, including frequent snacking, refined carbohydrate and sugar intake, and limited physical activity. These were addressed. However, the patient’s intense hunger occurred in the setting of symptomatic thyrotoxicosis. A physiologically increased drive to eat can be difficult to suppress and should not automatically be interpreted as willful nonadherence. Glimepiride was discontinued and replaced with pioglitazone. While this switch would not ordinarily be expected to produce greater immediate glucose lowering pharmacologically, reducing sulfonylurea exposure may also have lessened possible hypoglycemia-related hunger and thereby reduced frequent snacking and unhealthy carbohydrate intake. The degree and persistence of hyperglycemia nevertheless appeared disproportionate to the behavioral factors identified initially, prompting greater attention to the potential metabolic contribution of ongoing thyrotoxicosis.

The pre-insulin phase was particularly informative. Between November 2024 and May 2025, FPG improved from 306.5 mg/dL (17.0 mmol/L) to 183.4 mg/dL (10.2 mmol/L) and HbA1c from 11.1% (98 mmol/mol) to 8.9% (74 mmol/mol) while FT3 and FT4 also declined, despite withdrawal of glimepiride and before basal insulin had been introduced. When thyroid hormone excess subsequently became more marked in September 2025, glycemia deteriorated again. Later improvement occurred after both antithyroid treatment and progressive insulin intensification. Taken together, the longitudinal sequence supports thyrotoxicosis as a plausible contributor to metabolic instability, while the later insulin titration clearly also contributed to improved glycemia.

HbA1c requires additional context in thyrotoxicosis. It reflects glycemic exposure over the preceding weeks and therefore lags behind rapid changes in glucose or treatment. Hyperthyroidism can also increase erythrocyte turnover and produce an HbA1c that is lower relative to prevailing glycemia (5). For this reason, contemporaneous FPG values are particularly useful when interpreting short-term changes in this case.

### 3.2 Lipid pattern during thyrotoxicosis

The lipid findings provide a second clinically useful observation. Hyperthyroidism is associated with increased hepatic LDL-receptor activity and enhanced LDL clearance, and circulating PCSK9 may also decrease with thyroid hormone excess (3). In a systematic review and meta-analysis, treatment of overt hyperthyroidism was followed by increases in TC and LDL-C (4). The patient’s longitudinal pattern was compatible with this physiology: TC and directly measured LDL-C were low during more marked thyrotoxicosis and increased as thyroid hormone concentrations improved, despite the absence of statin or other lipid-lowering therapy.

Pioglitazone may also influence lipid measurements, but its characteristic effects are more prominent for triglycerides and high-density lipoprotein cholesterol. A meta-analysis of 15 randomized trials found a notable reduction in triglycerides and an increase in high-density lipoprotein cholesterol, with no significant overall change in LDL-C and only a small increase in TC (6). Pioglitazone therefore remains a relevant background influence, but its expected lipid profile does not readily account for the full longitudinal change observed in direct LDL-C and TC in this patient.

### 3.3 Blood pressure and propranolol

Hyperthyroidism lowers systemic vascular resistance while increasing heart rate, myocardial contractility, stroke volume, blood volume, and cardiac output, changes that can increase systolic blood pressure and widen pulse pressure (7). Blood pressure in this patient reached 160/90 mmHg during one period of marked biochemical thyrotoxicosis and subsequently declined, although the blood-pressure pattern was less consistent than the glycemic and lipid patterns.

Propranolol was used for adrenergic symptom control. Nonselective beta-blockade can alter the glycemic threshold for adrenergic warning symptoms of hypoglycemia and may blunt manifestations such as tremor and palpitations; delayed glucose recovery after insulin-induced hypoglycemia has also been described (8, 9). No documented hypoglycemic episodes occurred in this patient, making propranolol an important treatment consideration rather than a likely explanation for the major glycemic changes observed.

### 3.4 Clinical management and delayed thyroidectomy

The patient had already been diagnosed with toxic multinodular goiter before self-referring to our clinic, and her immediate goal was to improve diabetes control sufficiently to proceed with thyroid surgery. She preferred thyroidectomy as definitive treatment, and later compressive symptoms strengthened the clinical rationale for surgery. During follow-up, our clinic managed diabetes and thyrotoxicosis while she continued surgical follow-up at the other hospital. The surgical team requested better glycemic control before proceeding, and long intervals between surgical appointments because of high patient volume added a practical delay. Thyroidectomy had not been performed by the end of follow-up.

This created a prolonged period in which both endocrine disorders required active medical treatment. The patient’s experience also illustrates how metabolic control can influence access to definitive treatment for another endocrine condition, while symptoms from the untreated thyroid disorder continue to affect daily life.

### 3.5 Strengths and limitations

Strengths of this report include approximately 23 months of longitudinal follow-up, repeated FPG and thyroid measurements, directly measured LDL-C, visit-specific symptom documentation, serial blood-pressure measurements, detailed medication changes, and a patient perspective. The repeated pre-insulin and post-insulin observations allow the clinical sequence to be appreciated rather than reducing the case to a single before-and-after comparison.

This remains a single-patient observational case, and concurrent changes in diabetes treatment, diet, activity, and adherence prevent quantification of the independent contribution of thyroid status. Later adherence was based primarily on patient report, and systematic home-glucose downloads were unavailable. Detailed thyroid-ultrasound and thyroid-autoantibody records from the other hospital were not available for review, although the available radionuclide report provided a quantified uptake and supported the established diagnosis of toxic multinodular goiter. Thyroid-function testing was not available at every clinic visit, and there was no untreated comparison period.

## 4 Patient perspective

The patient described the period of symptomatic thyrotoxicosis and difficult diabetes control as a “roller-coaster.” She acknowledged that her diet was difficult to control initially, particularly because of intense hunger and frequent snacking. After counseling and medication changes, she described substantial efforts to follow medical advice but remained frustrated when glucose control continued to fluctuate.

During periods of greater thyrotoxic symptoms, she felt that she was “not herself” and described nervousness, irritability, severe insomnia, emotional instability, and withdrawal from family. When symptoms improved, she reported better sleep, less anxiety and irritability, and an improved sense of well-being. The continuing delay in thyroid surgery was an additional source of frustration because surgery was her preferred definitive treatment, yet it remained dependent on improving diabetes control and on availability of surgical appointments.

## 5 Conclusion

This longitudinal case shows how thyrotoxicosis may amplify metabolic instability in a patient with long-standing type 2 diabetes. Periods of greater thyroid hormone excess were accompanied by poorer glycemic control and lower TC and direct LDL-C, while improvement in thyroid status occurred alongside improvement in glycemia and rising lipid concentrations. The early pre-insulin improvement is particularly notable because it occurred after glimepiride had been replaced with pioglitazone and before basal insulin was introduced. The case also highlights an important clinical distinction: documented dietary difficulty does not exclude a physiological driver. Prominent hunger and increased food intake can be manifestations of thyrotoxicosis and may not be fully under voluntary control. Concurrent treatment changes mean that thyroid status cannot be assigned the entire metabolic effect, but the clinical course supports thyrotoxicosis as an important potential contributor rather than an incidental comorbidity. Clinicians should therefore avoid allowing an initial label of nonadherence to close diagnostic reasoning when deterioration appears disproportionate to behavioral factors. In this patient, poor glycemic control also delayed her preferred definitive treatment for toxic multinodular goiter.

## Data Availability

The original contributions presented in the study are included in the article/supplementary material. Further inquiries can be directed to the corresponding author.
